# Effects of Resistance Training on Change-of-Direction Speed in Youth and Young Physically Active and Athletic Adults: A Systematic Review with Meta-Analysis

**DOI:** 10.1007/s40279-020-01293-w

**Published:** 2020-05-25

**Authors:** Helmi Chaabene, Olaf Prieske, Jason Moran, Yassine Negra, Ahmed Attia, Urs Granacher

**Affiliations:** 1 Division of Training and Movement Sciences, Research Focus Cognitive Sciences, University of Potsdam, Am Neuen Palais 10, Building 12, 14469 Potsdam, Germany; 2 Division of Exercise and Movement, University of Applied Sciences for Sports and Management Potsdam, Potsdam, Germany; 3 School of Sport, Rehabilitation and Exercise Sciences, University of Essex, Colchester, UK; 4 Research Unit (UR17JS01) “Sport Performance, Health & Society”, Higher Institute of Sport and Physical Education of Ksar Saïd, University of “La Manouba”, Tunis, Tunisia

## Abstract

**Background:**

Change-of-direction (CoD) speed is a physical fitness attribute in many field-based team and individual sports. To date, no systematic review with meta-analysis available has examined the effects of resistance training (RT) on CoD speed in youth and adults.

**Objective:**

To aggregate the effects of RT on CoD speed in youth and young physically active and athletic adults, and to identify the key RT programme variables for training prescription.

**Data sources:**

A systematic literature search was conducted with PubMed, Web of Science, and Google Scholar, with no date restrictions, up to October 2019, to identify studies related to the effects of RT on CoD speed.

**Study Eligibility Criteria:**

Only controlled studies with baseline and follow-up measures were included if they examined the effects of RT (i.e., muscle actions against external resistances) on CoD speed in healthy youth (8–18 years) and young physically active/athletic male or female adults (19–28 years).

**Study Appraisal and Synthesis Methods:**

A random-effects model was used to calculate weighted standardised mean differences (SMD) between intervention and control groups. In addition, an independent single training factor analysis (i.e., RT frequency, intensity, volume) was undertaken. Further, to verify if any RT variable moderated effects on CoD speed, a multivariate random-effects meta-regression was conducted. The methodological quality of the included studies was assessed using the physiotherapy evidence database (PEDro) scale.

**Results:**

Fifteen studies, comprising 19 experimental groups, were included. The methodological quality of the studies was acceptable with a median PEDro score of 6. There was a significant large effect size of RT on CoD speed across all studies (SMD = − 0.82 [− 1.14 to − 0.49]). Subgroup analyses showed large effect sizes on CoD speed in males (SMD = − 0.95) contrasting with moderate improvements in females (SMD = − 0.60). There were large effect sizes on CoD speed in children (SMD = − 1.28) and adolescents (SMD = − 1.21) contrasting with moderate effects in adults (SMD = − 0.63). There was a moderate effect in elite athletes (SMD = − 0.69) contrasting with a large effect in subelite athletes (SMD = − 0.86). Differences between subgroups were not statistically significant. Similar improvements were observed regarding the effects of independently computed training variables. In terms of RT frequency, our results indicated that two sessions per week induced large effects on CoD speed (SMD = − 1.07) while programmes with three sessions resulted in moderate effects (SMD = − 0.53). For total training intervention duration, we observed large effects for ≤ 8 weeks (SMD = − 0.81) and > 8 weeks (SMD = − 0.85). For single session duration, we found large effects for ≤ 30 min and ≥ 45 min (both SMD = − 1.00). In terms of number of training sessions, we identified large effects for ≤ 16 sessions (SMD = − 0.83) and > 16 sessions (SMD = − 0.81). For training intensity, we found moderate effects for light-to-moderate (SMD = − 0.76) and vigorous-to-near maximal intensities (SMD = − 0.77). With regards to RT type, we observed large effects for free weights (SMD = − 0.99) and machine-based training (SMD = − 0.80). For combined free weights and machine-based training, moderate effects were identified (SMD = − 0.77). The meta-regression outcomes showed that none of the included training variables significantly predicted the effects of RT on CoD speed (*R*^2^ = 0.00).

**Conclusions:**

RT seems to be an effective means to improve CoD speed in youth and young physically active and athletic adults. Our findings indicate that the impact of RT on CoD speed may be more prominent in males than in females and in youth than in adults. Additionally, independently computed single factor analyses for different training variables showed that higher compared with lower RT intensities, frequencies, and volumes appear not to have an advantage on the magnitude of CoD speed improvements. In terms of RT type, similar improvements were observed following machine-based and free weights training.

## Key Points

**Table:** 

Change-of-direction speed is a fundamental physical attribute in many field- and court-based team (e.g., soccer, handball, rugby) and individual sports (e.g., combat sports, tennis).
Resistance training appears to be an effective means to improve change-of-direction speed in youth and young physically active and athletic adults.
It seems that sex and age categories can moderate the effects of resistance training on change-of-direction speed with youth and males showing larger adaptive potential than adults and females.
Results from independently computed single factor analyses for different training variables revealed that higher compared with lower resistance training volumes, frequencies, and intensities appear not to have an advantage on the magnitude of training-induced improvements in change-of-direction speed.

## Introduction

The ability to change direction while sprinting, also known as change-of-direction (CoD) speed, is a fundamental physical attribute in many field-based team (e.g., soccer, handball, rugby) [1, 2] and individual (e.g., combat sports, tennis) sports [3–5]. Skilled change-of-direction movements represent an athlete’s ability to decelerate as quickly as possible (i.e., braking phase) before rapidly re-accelerating (i.e., propulsive phase) in a new direction [1, 6]. Results from analyses of different sports have shown that CoD actions occur frequently (every 2–3 s) and repeatedly (> 1000/game) in soccer, tennis, rugby, and basketball [7]. Additionally, it has previously been demonstrated that CoD speed is effective in predicting on-field performance in American Football [8]. Further underlining its importance, CoD speed has been used to discriminate elite from sub-elite soccer players [2, 9–11], representing a practically relevant parameter for talent identification and selection [9, 11]. Accordingly, it is important to systematically develop CoD speed to increase sport performance and, ultimately, success in competition.

Several neural, biomechanical, anthropometric and musculoskeletal characteristics are associated with CoD speed [1, 6]. Leg muscle quality, which is an umbrella term for reactive strength, concentric strength and power, eccentric strength, and left-right muscle imbalance appears to be an important predictor of CoD speed [6]. Generally, resistance training (RT) is an effective way to improve these muscle qualities in different populations across the lifespan and particularly in youth and young adult athletes [12, 13]. This implies that RT-induced improvements in leg muscle quality may translate to CoD speed [14].

A number of published narrative reviews have reported testing-, training-, and performance-related issues associated with CoD speed [1, 2, 14]. For example, Brughelli et al. [14] summarised previous longitudinal studies that examined the effects of RT on CoD speed in recreationally active and athletic individuals. These authors reported no effect of strength and power training on CoD speed. However, it is noteworthy that most of the discussed protocols in the review of Brughelli et al. [14] lacked a control condition [15–18] making the inferences far from conclusive. In a systematic review with meta-analysis, Lesinski et al. [12] examined the effects of RT in young athletes on a wide range of measures of physical fitness, including CoD speed. These authors revealed that RT had moderate effects (standardised mean difference [SMD] = 0.68) on CoD speed. However, they did not provide specific training-related recommendations on how to prescribe RT to enhance CoD speed. Moreover, findings were limited to youth athletes aged six to 18 years. Additionally, both Brughelli et al. [14] and Lesinski et al. [12] adopted a broad definition of RT and included heterogeneous intervention programmes that used complex and/or plyometric training. Indeed, the effects of the latter on CoD speed have previously been meta-analysed [19, 20]. Specifically, Asadi et al. [20] demonstrated that plyometric training was effective in improving CoD speed (effect size [ES] = 0.96) in physically active and athletic populations. Similarly, in another meta-analysis, Asadi et al. [19] showed that plyometric training improved CoD speed (ES = 0.86) with a tendency towards greater training-related adaptations in more mature (mid [ES = 0.95] and postpubertal [ES = 0.99]), compared with less mature (prepubertal [ES = 0.68]), participants. Despite these findings, to date, no systematic review has examined the effects of RT (characterised by actions against external resistances [21, 22]) on CoD speed in youth and young physically active and athletic adults.

Considering the above, the question of how to appropriately prescribe RT to optimise CoD speed needs to be further clarified so that coaches can build more focused programmes for their athletes. In an attempt to fill this gap in the literature, the first aim of this systematic review with meta-analysis was to characterise the effects of RT on CoD speed in healthy youth and young physically active and athletic adults. The second aim was to identify the main RT programme variables that could be used for training prescription.

## Methods

This meta-analysis was conducted according to the preferred recording items for systematic review and meta-analyses (PRISMA) statements [23].

### Literature Search

**Fig. 1:**
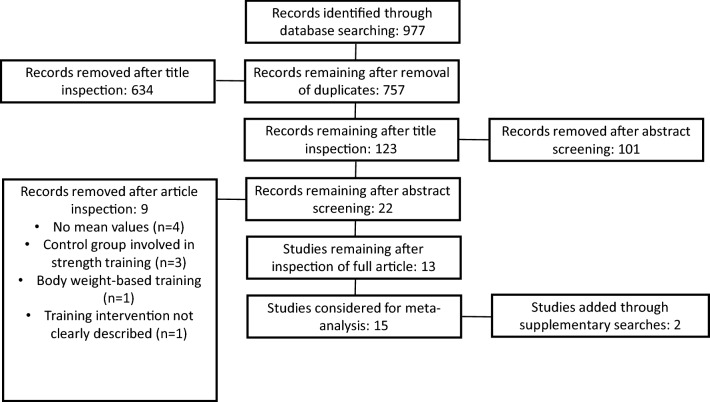
Flow chart of the included and excluded studies

A systematic search was conducted in PubMed, Web of Science, and Google Scholar with no date restrictions up to October 2019. Only controlled trials, which were peer-reviewed articles published in English, were considered. Keywords were collected through experts’ opinion, literature review, and controlled vocabulary (e.g., Medical Subject Headings [MeSH]). The following Boolean syntax was used: “resistance training”[Mesh] AND (“change of direction performance” OR “change of direction speed” OR agility) NOT (old OR elderly OR disease OR syndrome OR patient). Search results were screened by two researchers (HC and YN). In the process of selecting studies for inclusion, titles of all relevant articles were reviewed. Thereafter, abstracts and full texts were examined. Reference lists of review articles were manually searched for further potential studies that could be relevant for inclusion. An overview of the screening process is outlined in Fig. 1.

### Selection Criteria

A PICOS (participants, intervention, comparators, study outcomes, and study design) approach was used to rate studies according to their eligibility [23]. The following inclusion criteria were defined a priori. (1) population: a cohort of healthy youth and/or young physically active/athletic (male and/or female) adults, aged 8–28 years (2) intervention: resistance training intervention programme. With reference to previous studies [21, 22], RT was defined as “[requiring] the musculature to contract against an opposing force generated by some type of external resistance’’ [that is not bodyweight] (3) comparators: active or passive control groups (4) outcomes: at least one measure of CoD speed (e.g., *T* test time, Illinois CoD test time, 5-0-5 test time) with the performance outcome expressed in seconds (5) study design: controlled trials with baseline and follow-up measures. Exclusion criteria were defined a priori as follows: (1) a cohort with health problems (e.g., individuals with diabetes) (2) outside the predefined age range (3) RT programmes using body mass only (e.g., plyometric training) (4) RT programme combined with a non-external resistance (e.g., complex training) (5) no passive/active control group, and (6) lack of baseline and follow-up data in the study.

### Data Extraction

Data extraction was performed by two reviewers (HC, YN) using a standardised form created in Microsoft Excel. The first reviewer collected data and the second verified study eligibility. In the case of no agreement regarding data extraction and study eligibility, UG was consulted for clarifications. To calculate effect sizes, baseline and follow-up means and standard deviations for the aforementioned outcome measures were used. Of note, given that time (s) was the main outcome measure, negative effect size values were used to represent improvements in performance. The characteristics of the included studies are displayed in Tables 1 and 2.

**Table 1:** Characteristics of subjects from the included studies

Study	Study group	Sex	Sport	Age (years)	Body height (cm)	Body mass (kg)	Participants	Training status
Hammami et al. [48]	Exp	Male	Soccer	16.2 ± 0.6	175 ± 3	58.0 ± 6.2	16	Experienced level
	Cont (active)			16.8 ± 0.2	168 ± 5	58.1 ± 5.2	12	
Negra et al. [47]^a^	Exp	Male	Soccer	12.8 ± 0.3	159.3 ± 8.4	47.8 ± 6.8	12	Regional level
	Cont (active)			12.7 ± 0.3	153.2 ± 8.6	42.5 ± 5.5	11	
Torres-Torrelo et al. [63]	Exp	Male	Futsal	23.8 ± 2.4	177.2 ± 0.1	73.6 ± 7.0	12	Third division
	Cont (active)			24.7 ± 4.7	176.5 ± 0.1	75.9 ± 7.1	10	
Prieske et al. [45]	Exp 1	Male and female		22.6 ± 2.6	176.6 ± 8.7	73.5 ± 10.7	10	Physically active
	Exp 2			23.4 ± 3.2	178.2 ± 9	72.2 ± 9.6	9	
	Cont (active)			22.9 ± 2.4	174.9 ± 8	69.7 ± 10.1	16	
Negra et al. [46]^a^	Exp	Male	Soccer	12.8 ± 0.2	160.4 ± 9.1	49.2 ± 8.1	13	Regional level
	Cont (active)			12.7 ± 0.3	154.5 ± 11.1	45.4 ± 8.1	11	
Mcbride et al. [44]	Exp 1	Male	Various activities	24.2 ± 1.8	181.7 ± 3.5	84.4 ± 4.6	9	Club level
	Exp 2			21.6 ± 0.8	179.5 ± 2.0	80.5 ± 3.8	10	
	Cont (active)			22.3 ± 1.8	176.5 ± 3.0	79.1 ± 4.2	7	
Tricoli et al. [64]	Exp	Male	Various activities	22.0 ± 1.5	179.4 ± 8.8	73.4 ± 10.7	12	Recreational level
	Cont (passive)						8	
Christou et al. [43]^b^	Exp	Male	soccer	13.8 ± 0.4	162.0 ± 3.8	52.0 ± 3.3	9	Experienced level
	Cont (active)			13.5 ± 0.9	163.0 ± 2.5	54.1 ± 2.0	9	
Yildiz et al. [65]	Exp 1	Male	Tennis	9.6 ± 0.7	134.1 ± 6.8	31.3 ± 4.1	10	Recreational level
	Cont (active)						8	
Whitehead et al. [66]	Exp	Male		21.3 ± 1.8	177.3 ± 9.4	80.0 ± 2.6	10	
	Cont (passive)						10	
Kraemer et al. [67]	Exp 1 (Periodized)	Female	Tennis	19.2 ± 1.1	167.9 ± 5.6	60.5 ± 7.7	9	Experienced level
	Exp 2 (non-periodized)			18.6 ± 1.3	167.0 ± 4.1	60.8 ± 7.8	10	
	Cont (active)			19.3 ± 1.6	167.3 ± 6.1	60.1 ± 7.6	8	
Hammami et al. [68]^c^	Exp	Male	Soccer	16.2 ± 0.6	175.0 ± 3.0	58.1 ± 7.3	19	NR
	Cont (active)			15.8 ± 0.2	168.0 ± 5.0	58.2 ± 5.0	12	
Deane et al. [69]	Exp	Male	Various activities	21.2 ± 3.9	182.6 ± 6.8	–	13	Physically active
		Female		22.2 ± 3.9	164.9 ± 6.2	–	11	
	Cont (passive)	Male		21.4 ± 1.4	181.7 ± 6.8	–	11	
		Female		20.9 ± 2.8	164.5 ± 8.4	–	13	
Aloui et al. [70]	Exp	Male	Soccer	18.3 ± 0.8	184.0 ± 5.0	83.4 ± 17.0	15	Elite level
	Cont (active)			18.8 ± 0.8	185.0 ± 7.0	78.7 ± 13.8	15	
Barbalho et al. [71]	Exp	Male	Soccer	18.8 ± 0.8	178.4 ± 6.2	73.1 ± 6.6	12	Professional level
	Cont (active)			19.1 ± 0.9	176.3 ± 8.6	72.0 ± 5.9	11	

*NR* not reported, *Exp* experimental group, *Cont* control group

^a^Prepubertal

^b^Adolescent

^c^Pubertal

**Table 2:** Characteristics of the training interventions

Study	Study group	Number of weeks	Training frequency (per week)	Total training session	Session duration (min)	CoD test	Training programme	Training modality
Hammami et al. [48]	Exp	8	2	16	45	Sprint 4 × 5 m 9–3–6–3–9 m sprint with 180° turns	Back half squat (70–90% 1RM); 3–5 sets with 3–8 repetitions	Free weights
	Cont (active)						Regular soccer training	–
Negra et al. [47]^a^	Exp	12	2	24	35–40	Illinois CoD test	Back half squat (40–60% 1RM) using Smith machine; 4 sets with 10–12 repetitions	Machine-based
	Cont (active)						Regular soccer training	
Torres-Torrelo et al. [63]	Exp	6	2	12	20–25	25 m sprint with 45° CoD each 5 m	Full squat (2–3 sets with 4–6 repetitions at 1.2 m s^−1^ [~ 45% 1RM] to 1.0 m s^−1^ [~ 58% 1RM])	Free weights
	Cont (active)						Regular futsal training	–
Prieske et al. [45]	Exp 1	6	3	18	45–60	*T* test	Resisted linear sprint, heel-to-butt, knee lift, jump running, and lateral shuffle using elastic straps attached to a motorized treadmill (20–70 maximal sprint velocity, 3–5 kg expanders, 1.5–8% slope)	–
	Exp 2	6	3	18	45–60		Leg press, leg curl, knee extension, and calf raise (40% 1RM, 3–5 sets, 10 repetitions)	Machine-based
	Cont (active)						Regular physical activity	–
Negra et al. [46]^a^	Exp	12	2	24	80–90	*T* test Illinois CoD test	Back half squat (40–60% 1RM) using Smith machine; 4 sets with 10–12 repetitions	Machine-based
	Cont (active)						Regular soccer training	–
Mcbride et al. [44]	Exp 1	8	2	16		*T* test	Jump squats (5 sets at 30% 1RM; Number of repetitions per set determined by a decrease in PP output of 15%)	Machine-based
	Exp 2	8	2	16			Jump squats (4 sets at 80% 1RM; number of repetitions per set determined by a decrease in PP output of 15%)	Machine-based
	Cont (active)						Regular physical activity	
Tricoli et al. [64]	Exp	8	3	24		Agility test	Olympic weightlifting exercises: Half-squat (4 sets of 6RM); High-pull (3–4 sets of 6RM); power clean (4–6 sets of 4RM); clean and jerk (4–6 sets of 4RM)	Free weights
	Cont (passive)							
Christou et al. [43]^b^	Exp	16	2	32	45	10 × 5 m	Leg-press, bench-press, leg-extension, pec-deck, leg-flexion, overhead press, lat pull-downs, calf-raise, sit-ups, and upper-lower back-extension (55–80% 1RM, 2–3 sets with 8–15 repetitions)	Machine-based
	Cont (active)						Regular soccer training	
Yildiz et al. [65]	Exp 1	8	3	24	45–50	*T* test	Chest press, shoulder press, lateral pull-down, seated leg extension, leg curl, among others (3 sets with 10–12 repetitions)	Machine-based
	Cont (active)						Regular tennis training	–
Whitehead et al. [66]	Exp	8	2	16		5-0-5 CoD	Squat, leg press, leg extension, leg curl, lunges, calf raises (3 sets with 8 [80% 1RM] to 12 [75% 1RM] repetitions)	Combined machine-based and free weights
	Cont (passive)							
Kraemer et al. [67]	Exp 1 (Periodized)	16	3	NR	NR	Modified USTA-Agility test	Leg press, bench press, shoulder press, dumbbell lateral raise, dumbbell internal rotation, among others (2–3 sets of 4–15 RM)	Combined of free weight and machine-based exercises
		24	3	NR	NR			
		36	3	NR	NR			
	Exp 2 (non-periodized)	16	3	NR	NR		Leg press, bench press, shoulder press, dumbbell lateral raise, dumbbell internal rotation, among others (2–3 sets of 8–10 RM)	Combined of free weight and machine-based exercises
		24	3	NR	NR			
		36	3	NR	NR			
	Cont (active)							
Hammami et al. [68]^c^	Exp	8	2	16	30	Sprint test with 180° turns 9–3–6–3–9 m sprint with backward and forward running 4 × 5 m sprint with turns	Half-squat (70–90% 1RM) with 3–5 sets and 3–8 repetitions	Free weights
	Cont (active)						Regular soccer training	–
Deane et al. [69]	Exp	8	3	24	NR	4 × 5.8 m shuttle run	Hip flexion exercise using elastic tubing as a resistance tool (2 sets with 10 repetitions and a third set with maximal effort to failure)	Free weights
	Cont (passive)							
Aloui et al. [70]	Exp	8	2	16	30	Half *T* test	Knee extension and hip extension using elastic bands of various resistance; 3 sets with 12–15 repetitions	Free weights
	Cont (active)						Regular handball training	
Barbalho et al. [71]	Exp	15	3	45	NR	*T* test	Bench press, lateral pull down, shoulder press, leg press, free squatting with bar, seated leg curl, calf standing in the machine, among others (4–15 RM)	Combined machine-based and free weights
	Cont (active)						Regular soccer training	

*CoD* change-of-direction, *NR* not reported, *Exp* experimental group, *Cont* control group, *USTA* United States Tennis Association, *RM* repetition maximum, *PP* peak power

^a^Prepubertal

^b^Adolescent

^c^Pubertal

### Study quality

**Fig. 2:**
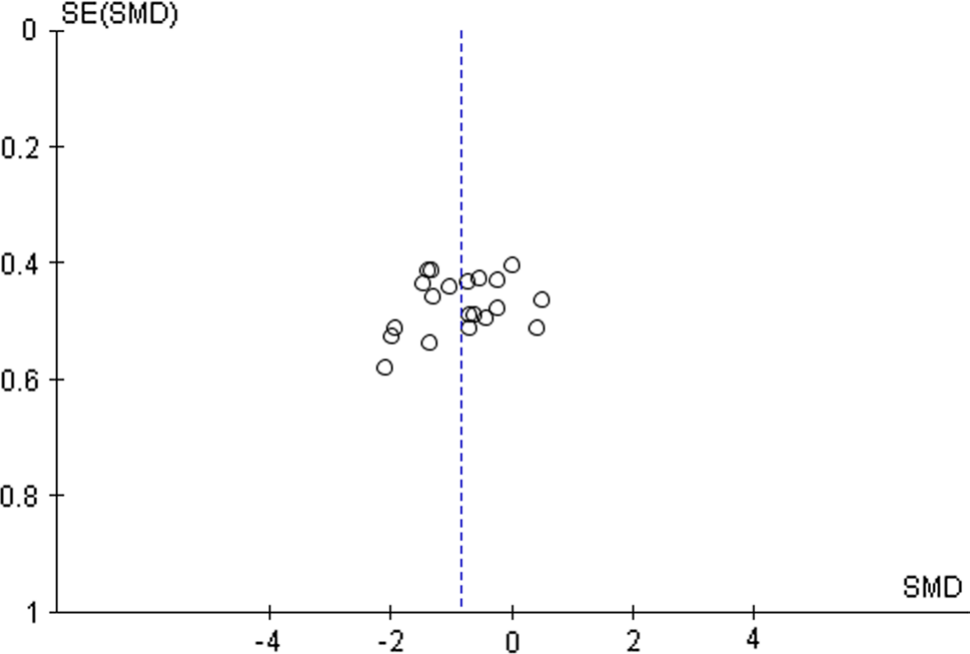
Funnel plot illustrating the symmetrical distribution of the effects across the included studies. *SMD* standardised mean difference, *SE(SMD)* standard error of the SMD

The risk of bias of the included studies was assessed using the Physiotherapy Evidence Database (PEDro) scale and the methodological quality of studies was rated on a scale from 0 (high risk of bias) to 10 (low risk of bias). A score of ≥ 6 represented the threshold for studies with low risk of bias [24] (Table 3). Further, funnel plots were generated by plotting SMDs against the standard error of the SMD (seSMD) to visually inspect for asymmetry and to examine the risk of publication bias (i.e., systematic heterogeneity) [25]. In a symmetrical funnel plot, the effects of smaller studies should scatter widely at the bottom, with the spread narrowing amongst the larger studies [26] (Fig. 2).

**Table 3:** Methodological quality of the included studies based on the physiotherapy evidence database (PEDro) scale

Study	Eligibility criteria	Randomized assignation	Blinded assignation	Group homogeneity	Blinded subjects	Blinded coaches	Blinded investigator	Dropout < 15%	Intention-to-treat	Group comparisons	Point and variability measures	Total PEDro score
Prieske et al. [45]	+	+	–	–	–	–	–	+	+	+	+	6
Torres-Torrelo et al. [63]	+	+	–	+	–	–	–	+	+	+	+	6
McBride et al. [44]	+	–	–	+	–	–	–	+	+	+	+	5
Hammami et al. [48]	+	+	–	+	–	–	–	+	+	+	+	6
Negra et al. [47]	+	+	–	+	–	–	–	+	+	+	+	6
Negra et al. [46]	+	+	–	+	–	–	–	+	+	+	+	6
Christou et al. [43]	+	–	–	–	–	–	–	+	+	+	+	4
Tricoli et al. [64]	+	+	–	–	–	–	–	–	+	+	+	4
Whitehead et al. [66]	+	–	–	–	–	–	–	+	+	+	+	4
Kraemer et al. [67]	+	+	–	+	–	–	–	+	+	+	+	6
Yildiz et al. [65]	+	–	–	+	–	–	–	+	+	+	+	5
Barbalho et al. [71]	+	+	–	–	–	–	–	+	+	+	+	5
Aloui et al. [70]	+	+	–	+	–	–	–	+	+	+	+	6
Deane et al. [69]	+	–	–	–	–	–	–	+	+	+	+	4
Hammami et al. [68]	+	+	–	+	–	–	–	+	+	+	+	6

The eligibility criteria have to be excluded for calculation of the total PEDro score; “+” = indicates a ‘‘yes’’ score; “−’’ = indicates a ‘‘no’’ score

### Results Analyses and Interpretation

To examine the effectiveness of RT on CoD speed performance, between-subject standardised mean differences (SMDs) were computed as follows:

$${\text{SMD}} = \frac{{{\text{Between - group}}\;{\text{mean}}\;{\text{outcome}}\;{\text{difference}}}}{{{\text{Pooled}}\;{\text{standard}}\;{\text{deviation}}\;{\text{of}}\;{\text{outcome}}\;{\text{among}}\;{\text{participants}}}}.$$

To control for sample size, SMDs were adjusted according to the following equation $\left( {1 - \frac{3}{4N - 9}} \right)$ [27] with N representing the total sample size. Quantitative data analysis was carried-out using RevMan version 5.3.5 [28]. A random-effects model was used to weight each study and to determine the SMDs which are presented alongside 95% confidence intervals. The SMDs were interpreted using the conventions as outlined by Cohen [29] (< 0.2 “trivial”; ≤ 0.2 SMD < 0.5 “small”, ≤ 0.5 SMD < 0.8 “moderate”, ≥ 0.8 “large”). When more than a single intervention group was included in a study, the control group was proportionately divided to facilitate comparison amongst all participants [30]. In addition, a multivariate random effects meta-regression was conducted with Comprehensive Meta-analysis version 3.3.70 (Biostat Inc., Englewood, NJ, USA) to verify if any of the training variables predicted the effects of RT on CoD speed.

The level of between-study heterogeneity was assessed using the *I*^2^ statistics. This indicates the proportion of effects that are caused by heterogeneity as opposed to chance [23]. Low, moderate, and high heterogeneity correspond to *I*^2^ outcomes of 25, 50, and 75%, respectively [31]. A value above 75% is rated as being considerably heterogeneous [32]. The *χ*^2^ (chi-square) statistic determines whether the differences in the results of the analysis are due to chance and in such a case, a low *p* value, or high *χ*^2^ statistic, relative to degrees of freedom would be apparent [32]. The level of significance was set at *p* < 0.05.

### Subgroup Analyses

Age was considered a moderator variable and participants were classified as a youth (8–18 years) and young adults (19–28 years). In the youth category, two sub-categories were considered: children (pre-pubertal, ≤ 13 years) and adolescents (mid- and post-pubertal, > 13 to ≤ 18 years) [33]. This range was used due to previously established maturation-related adaptations following RT [34]. Sex was chosen as a moderator variable due to potential RT-related differences in adaptations between males and females [35, 36]. Training expertise was another moderator variable due to differences in the magnitude of RT-related adaptations between elite athletes and athletes of lower expertise [37, 38]. Athletes competing at the national and international levels were considered to be elite [39].

### Single Factor Analyses

Single-factor analyses for training variables related to the applied RT programmes were conducted. For this purpose, we computed the effects of single training variables such as frequency (i.e., 2 vs. 3 sessions per week), session duration (i.e., ≤ 30 min vs. ≥ 45 min), total training duration (i.e., ≤ 8 vs. > 8 weeks intervention), total number of training sessions (i.e., ≤ 16 vs. > 16), and training intensity [40] on RT-related adaptations in CoD speed. In terms of training intensity, RT intensities between 30 and 69% of the one-repetition maximum (1RM) were considered light-to-moderate [41]. RT intensities ≥ 70% of the 1RM were considered vigorous-to-near maximal [41]. Training modality (i.e., free weights vs. machine-based training vs. combined free weights and machine-based training) was also included as a potentially moderating variable.

## Results

### The Methodological Quality of the Included Studies

The median PEDro score was six [95% confidence interval (CI) 4–6]. This score indicates an acceptable methodological quality (i.e., low risk of bias) of the included studies. Eight out of 15 studies achieved the threshold score of six on the PEDro scale (Table 3). The visual inspection of the funnel plots indicated no asymmetries and, hence, low risk of publication bias amongst the included studies.

### Main Effects

**Fig. 3:**
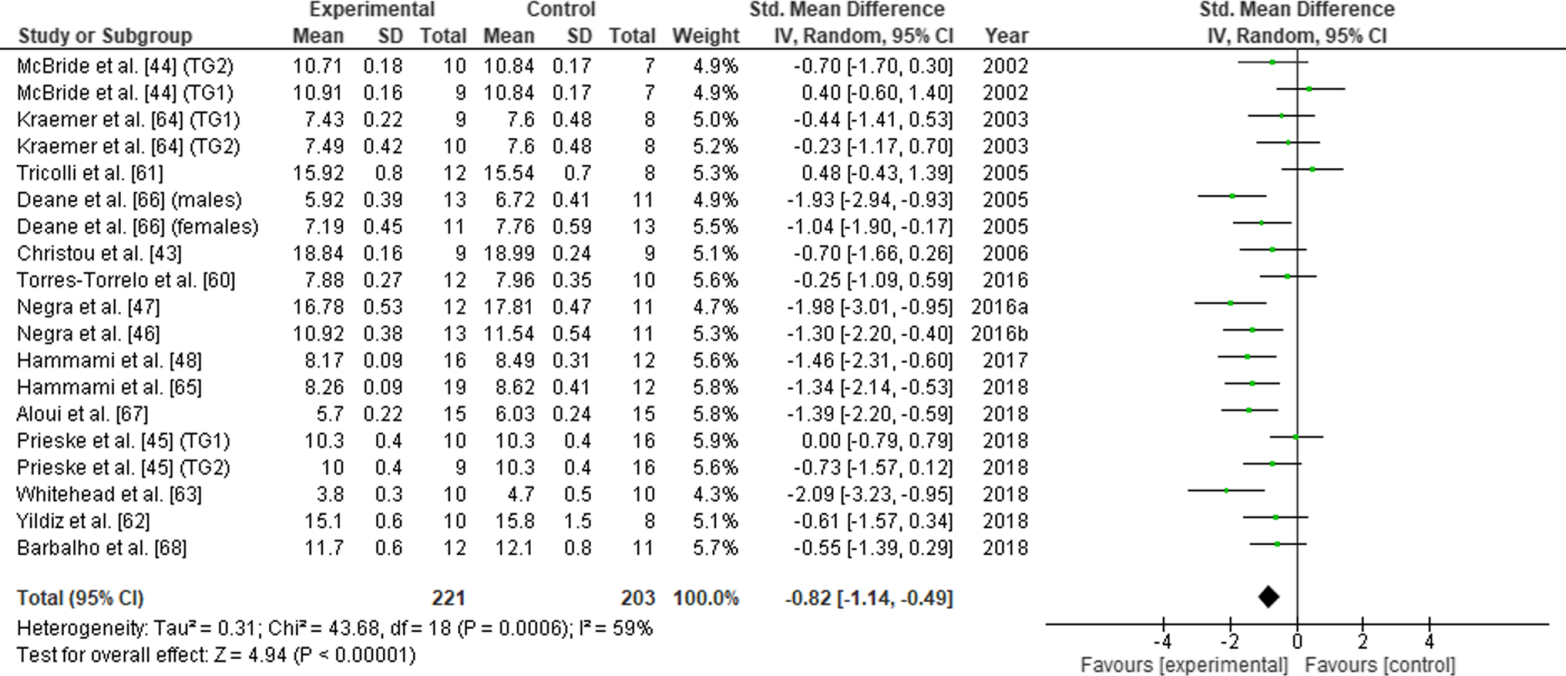
Effects of resistance training (experimental) versus active/passive control on change-of-direction speed performance. *TG* training group, *SD* standard deviation, *CI* confidence interval, *IV* inverse variance

A total of 15 studies were included in this meta-analysis. These studies consisted of 19 individual intervention arms which examined the effects of traditional RT (i.e., strength training with combined concentric and eccentric muscle actions) but not single-mode eccentric RT on measures of CoD. There was a significant positive effect of RT on CoD speed across all studies (SMD = − 0.82 [− 1.14 to − 0.49], *Z* = 4.94, *p* < 0.01) (Fig. 3). The overall effect was of large magnitude and showed a significant level of between-study heterogeneity (*I*^2^ = 59%, p < 0.001).

### Results of Subgroup Analyses

A summary of the effects of moderator variables is displayed in Table 4. The range of heterogeneity across the different subgroups extended from 0 to 70%. Although not significantly different (*p* = 0.31), males reached larger CoD speed adaptations (SMD = − 0.95; *p* < 0.01) following RT compared with females (SMD = − 0.60; *p* = 0.03), regardless of age. Children (SMD = − 1.28; *p* < 0.01) displayed similar CoD speed adaptations compared with adolescents (SMD = − 1.21; *p* < 0.01) and both groups showed greater adaptations compared with adult performers (SMD = − 0.63; *p* < 0.01). However, this trend was not statistically significant (*p* = 0.13). Despite this, large improvements in CoD speed were observed in children and adolescents whilst only a moderate enhancement was noted in the adult subgroup. Additionally, moderate CoD speed improvements following RT were observed in elite athletes (SMD = − 0.69; *p* < 0.01) whilst large increases were noted in subelite (recreationally active or regional level) athletes (SMD = − 0.89; *p* < 0.01). However, this difference was not statistically significant (*p* = 0.61).

**Table 4:** Subgroup and single training factor analyses

Subgroup	Studies (*n*)	Participants (n)	Estimated effect size Mean (95%, CI)	Within group* p*	Between group* p*	Within group *I*^2^ (%)	Effect descriptor
Sex							
Male	14	314	– 0.95 (– 1.36 to – 0.53)	< 0.01	0.31	64	Large
Female	3	59	– 0.60 (– 1.13 to – 0.07)	0.03		0	Moderate
Age groups							
Children (≤ 13 years)	3	65	– 1.28 (– 2.02 to – 0.53)	< 0.01	0.13	45	Large
Adolescents (> 13 to ≤ 18 years)	3	73	– 1.21 (– 1.71 to – 0.71)	< 0.01		0	Large
Young adults (19–28 years)	13	282	– 0.63 (– 1.04 to – 0.22)	< 0.01		61	Moderate
Training experience							
Elite	4	88	– 0.69 (– 1.21 to – 0.17)	< 0.01	0.61	28	Moderate
Recreational/active/regional level	15	336	– 0.86 (– 1.26 to – 0.47)	< 0.01		64	Large
Training frequency							
2 sessions/week	10	229	– 1.07 (– 1.52 to – 0.62)	< 0.01	0.09	58	Large
3 session/week	9	195	– 0.54 (– 0.97 to – 0.12)	≤ 0.01		50	Moderate
Training duration							
≤8 weeks	13	301	– 0.81 (– 1.23 to – 0.38)	< 0.01	0.91	66	Large
>8 weeks	6	123	– 0.85 (– 1.34 to – 0.35)	< 0.01		41	Large
Training type							
Machine-based training	7	141	– 0.80 (– 1.31 to – 0.29)	< 0.01	0.87	50	Large
Free weights	7	179	– 0.99 (– 1.57 to 0.40)	< 0.01		69	Large
Combined machine-based and free weights training	4	78	– 0.77 (– 1.51 to – 0.03)	< 0.05		58	Moderate
Session duration							
≤ 30 min	3	83	– 1.00 (– 1.72 to – 0.28)	< 0.01	0.99	57	Large
≥ 45 min	10	226	– 1.03 (– 1.53 to – 0.47)	< 0.01		69	Large
Total number of training sessions							
≤ 16	9	215	– 0.83 (– 1.32 to – 0.33)	< 0.01	0.97	64	Large
> 16	10	209	– 0.81 (– 1.27 to – 0.36)	< 0.01		58	Large
Training intensity							
≥ 30 to < 69% 1RM	5	110	– 0.76 (– 1.51 to – 0.01)	< 0.01	0.87	70	Moderate
≥ 70% 1RM	9	192	– 0.77 (– 1.24 to – 0.29)	< 0.01		57	Moderate

*CI* confidence interval, *n* number, *RM* repetition maximum

### Results of Single Training Factor Analyses

Although not statistically significant (*p* = 0.09), RT interventions including two sessions per week induced larger CoD speed improvements (SMD = − 1.07; *p* < 0.01) than three sessions per week (SMD = − 0.54; *p* < 0.05). Additionally, RT interventions that lasted ≤ 8 weeks or > 8 weeks induced similarly large effects on CoD speed (≤ 8 weeks: SMD = − 0.81; *p* < 0.01; > 8 weeks: SMD = − 0.85; *p* < 0.01) with no significant differences between subgroups (*p* = 0.91). Considering RT type, similar large effects following machine-based training (SMD = − 0.80; *p* < 0.01) and free weights training (SMD = − 0.99; *p* < 0.01) were observed on CoD speed. In terms of combined free weights and machine-based training, a moderate effect was noted (SMD = − 0.77; *p* < 0.05). The difference between the three training modalities was not statistically significant (*p* = 0.87). Furthermore, session durations of ≤ 30 min (SMD = − 1.00; *p* < 0.01) or ≥ 45 min (SMD = − 1.00; *p* < 0.01) showed similarly large effects on CoD speed. Again, the difference between ≤ 30 min and ≥ 45 min was not statistically significant (*p* = 0.99). In terms of the total number of training sessions, no significant differences were observed between ≤ 16 or > 16 sessions (*p* = 0.97). In both cases, the RT effect was of large magnitude (≤ 16: SMD = − 0.83; *p* < 0.01; > 16: SMD = − 0.81; *p* < 0.01). Considering training intensity, light-to-moderate intensities (≥ 30% to < 69% 1RM) (SMD = − 0.76; *p* = 0.05) were as effective as vigorous-to-near maximal (≥ 70% 1RM) (SMD = − 0.77; *p* < 0.01) with no significant difference between the two ranges (*p* = 0.87).

### Results of the Meta-Regression

The random effects meta-regression included four training variables (i.e., the total duration of the training, training frequency, training intensity, and session duration) (Table 5). None of the training variables significantly predicted the effects of RT on CoD speed (*p* = 0.61–0.96). Additionally, the meta-regression analysis featured a coefficient of determination of *R*^2^ = 0.00.

**Table 5:** Outcomes of the multivariate random-effect meta-regression for training variables to predict RT effects on CoD speed performance in youth and young physically active and athletic adults

Training variables	Coefficient	Standard error	95% CI	*Z* value	Two-sided *p*-value
Intercept	– 1.5058	2.9525	– 7.29 to 4.28	– 0.51	0.6101
Total duration of training	– 0.0055	0.1246	– 0.25 to 0.24	– 0.04	0.9651
Training frequency	0.4208	1.1159	– 1.77 to 2.61	0.38	0.7061
Session duration	– 0.0084	0.022	– 0.05 to 0.03	– 0.38	0.7029
Training intensity	– 0.1203	0.7553	– 1.60 to 1.36	– 0.16	0.8735

*RT* resistance training, *CoD* change-of-direction, *CI* confidence interval

## Discussion

CoD speed represents an important and practically relevant performance determinant in a wide range of team (e.g., soccer, handball, rugby, etc.) [1, 2] and individual (e.g., combat sports, tennis) sports [3–5]. Our findings revealed that RT is effective in improving CoD speed in youth and young physically active and athletic adults. In addition, these results demonstrate greater RT-related effects in males compared with females and in younger (i.e., children and adolescents) compared with older (i.e., adult) individuals. However, the observed differences between subgroups were not statistically significant. Independently computed single factor analyses for different training variables (i.e., frequency [2 vs. 3 sessions per week]; duration [≤ 8 vs. > 8 weeks intervention]; session duration [≤ 30 min vs. ≥ 45 min]; total number of RT sessions [≤ 16 vs. > 16]; and RT intensity [light-to-moderate vs. vigorous-to-near maximal]) and type (i.e., free weights vs. machine-based vs. combined free weights and machine-based training) indicated that there is no particular advantage of any single training variable or type over the other.

### Main Effects

Several factors associated with leg muscle qualities (i.e., reactive strength, concentric/eccentric strength, and left-right muscle imbalance) have been suggested to be among the primary determinants of CoD speed [1, 42]. Chaabene et al. [6] previously indicated that to rapidly decelerate and accelerate the human body during CoD tasks, it is crucial to systematically develop eccentric (deceleration) and concentric (acceleration) strength of the lower limbs. The main finding of the present review indicated large positive effects of RT on CoD speed, regardless of sex, age, and training expertise. This suggests that the improvement of leg muscle qualities, particularly lower limb muscle strength, can transfer directly to CoD speed. This is in line with previously conducted controlled trials showing moderate-to-large effects of RT on CoD speed in young individuals [43–48]. For instance, Christou et al. [43] studied the effects of strength training on CoD speed in adolescent soccer players and observed performance improvements of approximately 3% after 8 weeks and 5% after 16 weeks of training. Likewise, Hammami et al. [48] examined the effects of RT on CoD speed in male soccer players aged 16 years and demonstrated significant gains (2–4%) after 8 weeks of training. Moreover, it has been shown that there are greater strength levels in individuals who are faster during CoD tests, compared with those who are slower [49, 50]. Overall, RT interventions that target lower limb muscle strength appear to be effective in enhancing CoD speed in youth and young physically active and athletic adults. The observed RT-related improvements in CoD speed could be caused by a combination of neural and morphological factors. In terms of neural factors, higher levels of motor unit recruitment and synchronization, as well as rate coding (firing frequency), may be among the main mechanisms that contribute to better CoD speed (39). These physiological mechanisms may enable more effective and efficient activation of the stretch-shortening cycle [51]. Regarding morphological factors, an increase in the size of muscle fibres and, therefore, the level of force that a muscle and/or group of muscles can deliver, after RT could result in higher CoD speed performance [2]. It is well known that high levels of strength and power (rate of force development) are critical for performance in CoD speed tasks [13]. In light of this, RT has been shown to be effective in improving strength and power performances [12, 13, 37] and, by extension, CoD speed.

### Subgroup Analyses

Subgroup analyses indicated no significant sex differences with regards to the effects of RT on CoD speed. Regardless of this, it must be noted that the magnitude of the effect size was substantially larger in males (SMD = − 0.95) than it was in females (SMD = − 0.60). This aligns with the literature as it has been reported that males and females adapt differently to RT [35, 36]. These difference in the level of adaptations could be caused by sex-related differences in circulating anabolic hormones [52, 53] which are elevated in males compared with females from puberty. Maturation-related changes in the hormonal system have an impact on muscle hypertrophy [53, 54]. For example, during the adolescent growth spurt, it has been demonstrated that males gain up to 7.2 kg of muscle mass per year while females gain 3.5 kg over the same time period [55]. Indeed, females experience a larger increase in fat mass (inactive tissue) than muscle mass (active tissue) during this key developmental period [56]. These maturational changes regulate adaptive responses to RT leading to lower training-related adaptations in females compared with males. Moran et al. conducted two meta-analyses dealing with the effects of RT on strength performance in young males [34] and females [40]. These authors revealed that males [34] achieved large strength adaptations following RT (SMD = 0.98) while females displayed only moderate improvements (SMD = 0.54) [40]. It is well-established that muscular strength represents an important performance determinant for CoD speed [1, 6]. Accordingly, the observed greater training-related CoD speed improvements in males compared with females could partly be attributed to larger RT-related increases in muscle strength. Of note, there are fewer studies available that examined the effects of RT in females compared with males which is why we retrieved only three eligible studies that were conducted with females. This means that the outcome of the between-sex comparison must be interpreted with caution. Accordingly, future studies are warranted to examine the effects of RT on CoD speed in females.

Age was also considered a moderator because of previously established RT-related differences in adaptations between the various maturity stages [34]. Though chronological age is not directly aligned with maturation, older youth tend to be more mature than their younger counterparts [34]. Based on a previous study [33], participants were classified as children (pre-pubertal, ≤ 13 years), adolescents (mid- and post-pubertal, > 13 to ≤ 18 years), and young adults (> 18 years). Our findings showed similar RT-related CoD speed improvements between children (SMD = − 1.28) and adolescents (SMD = − 1.21). However, when compared with adults (SMD = − 0.63), children and adolescents achieved larger adaptations. Converse to this, Moran et al. [40] showed greater RT-related strength adaptations in older (> 15 years, ES = 0.72), compared to younger, (< 15 years, ES = 0.38) females. Likewise, in another meta-analysis examining the effects of RT on muscle strength in males, Moran et al. [34] demonstrated larger strength gains in pubertal (ES = 1.11) and postpubertal (ES = 1.01) compared with prepubertal individuals (ES = 0.50). The authors attributed the lower magnitude of RT-related strength adaptations to the fewer pathways of adaptation in younger (i.e., mostly neural in nature) compared to older participants (i.e., neural and morphological factors) [34]. Asadi et al. [19] showed that plyometric training improved CoD speed (ES = 0.86) with a tendency towards greater training-related adaptations in more mature (mid [ES = 0.95] and postpubertal [ES = 0.99]) compared with less mature (prepubertal [ES = 0.68]) participants. The cause of this divergence between the outcomes of the present study and previous studies [19, 34, 40] is not clear but it seems that other factors could have moderated the effects of RT on CoD speed. For instance, the main outcome differences between the present review (a task of relative strength and high coordinative level) and the previous research [34, 40] (task affording maximal strength) appear to have reversed the trend of adaptation. Moreover, categorising maturation was done in a slightly different way compared with previous studies [34, 40]. Specifically, the study of Moran et al. [34] does not include young adults category and considered mid and pubertal individuals in two separate categories. In the current study, the mid and pubertal subjects were merged into one single category. This could have affected the results. Also, the diminished resistance training-related adaptations observed in older compared with younger individuals can most likely be explained by the law of diminishing returns. Specifically, to achieve similar performance gains, individuals with high fitness status and/or more training experience (i.e., the time span an individual has been performing RT) have to increase their training volume more than individuals who are less fit and/or less experienced [57]. In fact, it has been shown that when the applied training stimulus is similar, individuals with more training experience are less likely to achieve large magnitudes of performance improvements compared with less experienced subjects [37, 56]. This hypothesis is reinforced by the training status of the adult participants that were included in this meta-analysis. In fact, the training status ranged from physically active individuals to elite level athletes. Notably, the majority of participants were included in structured sports activities. We consider our maturity-specific findings preliminary given the limited studies that have been conducted with children (*n* = 3) and adolescents (*n* = 3), compared to adults (*n* = 13). Accordingly, more research is needed to examine the effects of RT on CoD speed adaptations according to biological maturity level. Additionally, given that the physiology of males and females varies with age and maturation [52, 58], further studies exploring sex-specific CoD speed adaptations following RT are needed, taking biological maturity into consideration.

In terms of the level of competitiveness of the participants, results demonstrated moderate CoD speed improvements to RT in elite athletes and large increases in sub-elite athletes. As outlined above, it has been reported that gains in any RT-related measure are affected by the degree of adaptation that has already been realised by a trainee [57, 59], implying the presence of a ceiling preventing continued adaptation. Indeed, a lower magnitude of adaptation would be expected in individuals with more, compared with less, training experience [56], explaining the larger RT-related adaptations in CoD speed in sub-elite compared with elite athletes.

### Effects of Single Training Factors

Our findings showed larger RT-related CoD speed adaptation magnitude following two (SMD = − 1.07), compared with three, RT sessions per week (SMD = − 0.54). However, this was not statistically significant. For the remaining variables (i.e., total training intervention duration and single session duration, number of training sessions, and training intensity), similar improvements were observed. Moran et al. [40] revealed larger RT-related adaptations in strength performance in studies with fewer training sessions (≤ 16), shorter training intervention duration (≤ 8 weeks) and lower training frequencies (≤ 2 sessions per week) in females. Similar to what we observed, Asadi et al. [20] demonstrated no additional effects of high, compared to moderate, and low, intensity plyometric training on CoD speed. Overall, based on our findings, it seems more beneficial to favour lower over higher RT frequencies, volumes, and intensities to improve CoD speed in youth and young physically active and athletic adults. This may allow more time for sport-specific technical and tactical training and for the trainee to recover and adapt to the applied training stimuli [60]. Regarding RT type, our results indicate large-sized effects for free weights (SMD = − 0.99) and machine-based training (SMD = − 0.80) and moderate effects for combined free weights and machine-based training (SMD = − 0.77). Of note, there were no significant differences between the three training modalities. Lesinski et al. [12] studied the effects of RT on components of physical fitness in young athletes. These authors revealed that RT interventions using free weights were more effective in improving muscle strength (SMD = 2.97) and CoD speed (SMD = 1.31) than other training modalities such as machine-based training and combined free weights and machine-based training. However, these findings are limited by the small number of studies that were included in each subanalysis. For example, for muscle strength, Lesinski and colleagues included three studies using machine-based RT and only two studies using RT with free weights [12]. For CoD speed, only one study used free weights RT and none used machine-based RT. Future research is needed that directly contrasts the effects of machine-based versus free weights RT on CoD speed. Eccentric RT has been shown to play a crucial role during CoD tasks, particularly during the braking (deceleration) phase [6]. However, we should mention that none of the included studies in this meta-analysis have examined the effects of eccentric RT. This raises a gap in the literature indicating that future studies are required to cover this promising topic.

### Limitations

The present systematic review with meta-analysis has some limitations that warrant discussion. First, all the included studies examined the effects of RT on so-called ‘pre-planned’ CoD speed tests where no immediate reaction to a stimulus was required of the participant. Indeed, in an organic sports setting, directional changes are generally performed in response to an external stimulus (i.e. movement of opponent or ball) [1] and this means that in addition to the physical component of CoD, a cognitive component (e.g., recognition of a stimulus, reaction) is also demanded. To the authors’ knowledge, there are no studies available in the literature that examined the effects of RT on pure agility performance (i.e. physical and cognitive components) [2]. The few intervention studies available investigated the effects of small-sided games or video-based perceptual training on agility performance, among others [2]. This seems to be due to the complex nature of agility performance which is dependent on both the cognitive (decision making) and physical (e.g., acceleration/deceleration, etc) components of movement and performance [1]. Moreover, there appears to be a distinct lack of well-accepted and valid assessment protocols for agility as characterised by these observations. Of note, it has previously been postulated that the relevant importance of muscular strength may diminish if a cognitive challenge is included [2]. This is reinforced by a previous cross-sectional study showing trivial-to-small associations between agility and measures of physical fitness including muscle strength [50]. However, the question of the effects of RT on agility performance is yet to be explored and should be the focus of future studies. This could help to develop more ecologically valid training-related recommendations. A second limitation is related to the limited number of eligible studies and, particularly, the significant heterogeneity across the included studies which undermines the accuracy of the inter-study comparison. Moreover, for subgroup analyses, the dichotomisation of continuous data could result in residual confounding and reduced statistical power [61, 62].

## Conclusions

RT seems to be an effective means to improve CoD speed in youth and young physically active and athletic adults. Regarding sex differences, our subgroup analyses showed that males achieved larger adaptations than females, though the differences were not statistically significant. Additionally, we noted greater RT-related CoD speed improvements in children and adolescents, compared with adults. This seems to be related to the lower training age in younger, compared to older participants which might negate other relevant factors such as fewer available pathways of adaptation. Furthermore, independently computed single factor analyses for different training variables showed that higher compared with lower RT volumes, frequencies, and intensities had no advantage in the magnitude of CoD speed improvements. This finding can be explained by the principle of training specificity and how it applies to the force-velocity relationship. In other words, high-speed movements as in CoD tasks afford strength exercises of low resistance and high movement speed. However, this hypothesis needs to be verified in future studies. Regarding RT type, comparable improvements in CoD speed were achieved following either free weights or machine-based training. This finding needs to be substantiated in future longitudinal studies.
